# Adenoviruses Associated with Acute Diarrhea in Children in Beijing, China

**DOI:** 10.1371/journal.pone.0088791

**Published:** 2014-02-12

**Authors:** Liying Liu, Yuan Qian, You Zhang, Jie Deng, Liping Jia, Huijin Dong

**Affiliations:** Laboratory of Virology, Capital Institute of Pediatrics, Beijing, China; Queen Mary University of London, United Kingdom

## Abstract

Adenoviruses have been recognized as important causal pathogens of community-acquired diarrhea (CAD) among children, but their role in hospital-acquired diarrhea (HAD) is not well-understood. Hospitalized children with acute diarrhea and children who visited the outpatient department due to diarrhea were investigated from 2011 to 2012. Adenovirus was detected in stool specimens by PCR and further characterized by sequencing and phylogenetic analysis. SPSS software (version 19.0) was used for statistical analyses. A total of 2233 diarrheal children were enrolled in this study; this sample was comprised of 1371 hospitalized children, including 885 with CAD (IP-CAD) and 486 with HAD, and 862 outpatients with CAD (OP-CAD). Among these 2,233 patients, adenovirus was detected in 219 cases (9.8%). The positive rates for adenovirus were significantly different between the IP-CAD (9.3%), HAD (13.8%) and OP-CAD (8.1%) cases (X^2^ = 11.76, p = 0.003). The positive rate of adenovirus was lower in infants under six months of age compared to the positive rates in the other age groups. Of the 219 of adenovirus positive patients, 91 (41.6%) were identified as having serotype 41. Although enteric adenovirus (group F) was the most frequently detected adenovirus among children with either CAD or HAD, the role of non-enteric adenoviruses, especially the adenovirus 31 type (19.7%), cannot be ignored in diarrheal children.

## Introduction

Acute diarrhea is one of the most common diseases in infants and young children worldwide. Adenovirus (Ad), as well as rotavirus and norovirus, is an important causal pathogen in childhood diarrhea [1]. Ad usually accounts for 3.2 to 12.5% of acute diarrhea cases, and the detection ratio is higher in developing countries than in developed countries [2], [3], [4]. Most studies related to Ad-associated diarrhea in children have focused on community-acquired diarrhea (CAD) and on group F Ad, which is termed enteric Ad (EAd) and caused by the Ad serotypes 40 (Ad40) and 41 (Ad41) [5], [6], [7]. The importance of hospital-acquired infection (HAIs) (i.e., infections that become clinically evident after 48 hours of admission) has been highlighted in several recent publications [8], [9], [10], [11]. These publications have improved our understanding of the viral pathogenicity and epidemiology of HAI. The data about Ad in children with hospital-acquired diarrhea (HAD) obtained by our research team in 2010 indicate that Ad is one of the important pathogens of HAD and that some types of Ad, in addition to the F group, can cause infantile and childhood diarrhea [12]. However, our previous study, similar to other studies [10], [11], suffers the limitations of a small cohort size and a short investigation period. To increase our understanding of the incidence and epidemiology of Ad in children with acute diarrhea, we continued to investigate relationship between Ad infection and acute diarrhea in hospitalized children from 2011 to 2012. Additionally, children who visited the outpatient department for acute diarrhea that was classified as CAD were simultaneously tested over the same period.

## Materials and Methods

### Ethics Statement

This project was approved by the Ethics Committee of the Capital Institute of Pediatrics (No. 2012004). Written informed consent was not required because the patients enrolled in this study were fully anonymized, and the stool specimens used for this study were surpluses from routine laboratory tests. Verbal informed consent was obtained from the parents or guardians, and this was sufficient for approval by the ethics committee of the Capital Institute of Pediatrics. This consent was recorded by two staff members involved in the study (a clinician and a researcher) who explained the study procedures.

### Study Setting

The study was conducted at the Children's Hospital affiliated with the Capital Institute of Pediatrics. The hospital has 400 inpatient beds and receives approximately 1.5 million outpatient visits and 13 thousand inpatients who undergo approximately 2,700 operations annually.

### Collection of Fecal Specimens

Acute diarrhea was defined as ≥3 loose, or looser-than-normal stools in a 24-hour period and significant changes to the fecal exterior that included watery textures, mucous, or thin paste but excluded the presence of pus or blood (i.e., white cell counts ≤5/hpf and red cell counts = 0/hpf). Children younger than 6 years old who were admitted to the hospital or visited the outpatient department due to acute diarrhea were enrolled in this study. Hospitalized children with acute diarrhea included children having CAD who developed diarrhea within 48 hours of hospital admission and children having HAD whose symptom of diarrhea occurred over 48 hours after hospitalization [10]. Among the children who visited the outpatient department, acute diarrhea was defined as CAD. Thus, the children with acute diarrhea in this study were classified into the following three groups: hospitalized children with CAD (also called inpatients with CAD, IP-CAD), hospitalized children with HAD (HAD) and outpatient children with CAD (OP-CAD).

Stool specimens were collected between January 1, 2011 and December 31, 2012. After collection, these stool samples were held at room temperature for no more than four hours before freezing and were kept frozen no longer than two weeks before testing.

### Virus Detection

Nucleic acid was extracted from fecal specimens using DNAzol (Molecular Research Center, Inc., Cincinnati, Ohio, USA) and the method that we have previously published [13]. Ad DNA was detected with conventional polymerase chain reactions (PCRs) using the universal primers for all types of Ad [14], [15] and followed by PCR with specific primers [16] for the identification of Ad group F (Ad 40 and Ad 41) (Table 1). PCR was performed under conditions identical to those described previously [15], [16]. The PCR products were verified by sequencing and then analyzed at the NCBI website (http://www.ncbi.nlm.nih.gov/blast/Blast.cgi). The specimens were also tested for rotavirus using the Rota/Adenoscreen Dipstick (Microgen Bioproducts Ltd., Camberley, Surrey, UK). The sequences of Ad 41 that were amplified from fecal specimens were aligned with CLUSTAL W software. The neighbor-joining (NJ) algorithm in MEGA4 was used for phylogenetic analyses. Genetic distances were calculated by the Jukescantor parameter, and the reliabilities of phylogenetic trees were examined with bootstrap tests with 1,000 replications.

**Table 1:** Primers for Ad identification.

Target genes	Primers	Position	sequences(5′-3′)	length(nt)
Hexon	hexAA1885(+)	21–45	GCCSCARTGGKCWTACATGCACATC	301
	hexAA1913(−)	321–301	CAGCACSCCICGRATGTCAAA	
Fiber	fibF1(+)	396–426	ACTTAATGCTGACACGGGCAC	541(Ad40)
	fibF2(−)	1006–1028	TAATGTTTGTGTTACTCCGCTC	586(Ad41)

note: R = A/G, K = G/T, S = C/G, W = A/T, I = Hypoxanthine.

### Data analyses

The medical records of all enrolled children were reviewed, and information, including gender, age, the date of fecal sample collection, and the identities of the wards in which the specimens were collected, was recorded. Statistical analyses were performed with SPSS software (version 19.0, SPSS Inc., USA). Chi-square (**χ^2^**) tests were used to compare the means across groups, and differences were considered significant when **p** was less than 0.05.

## Results

### Diarrhea Classification

Of the 1,371 hospitalized children with acute diarrhea in this study, 885 were classified as IP-CAD, and 486 were classified as HAD. Additionally, 862 cases were classified as OP-CAD because these cases of acute diarrhea were identified in the outpatient department.

### Ad detection rates

**Figure 1:**
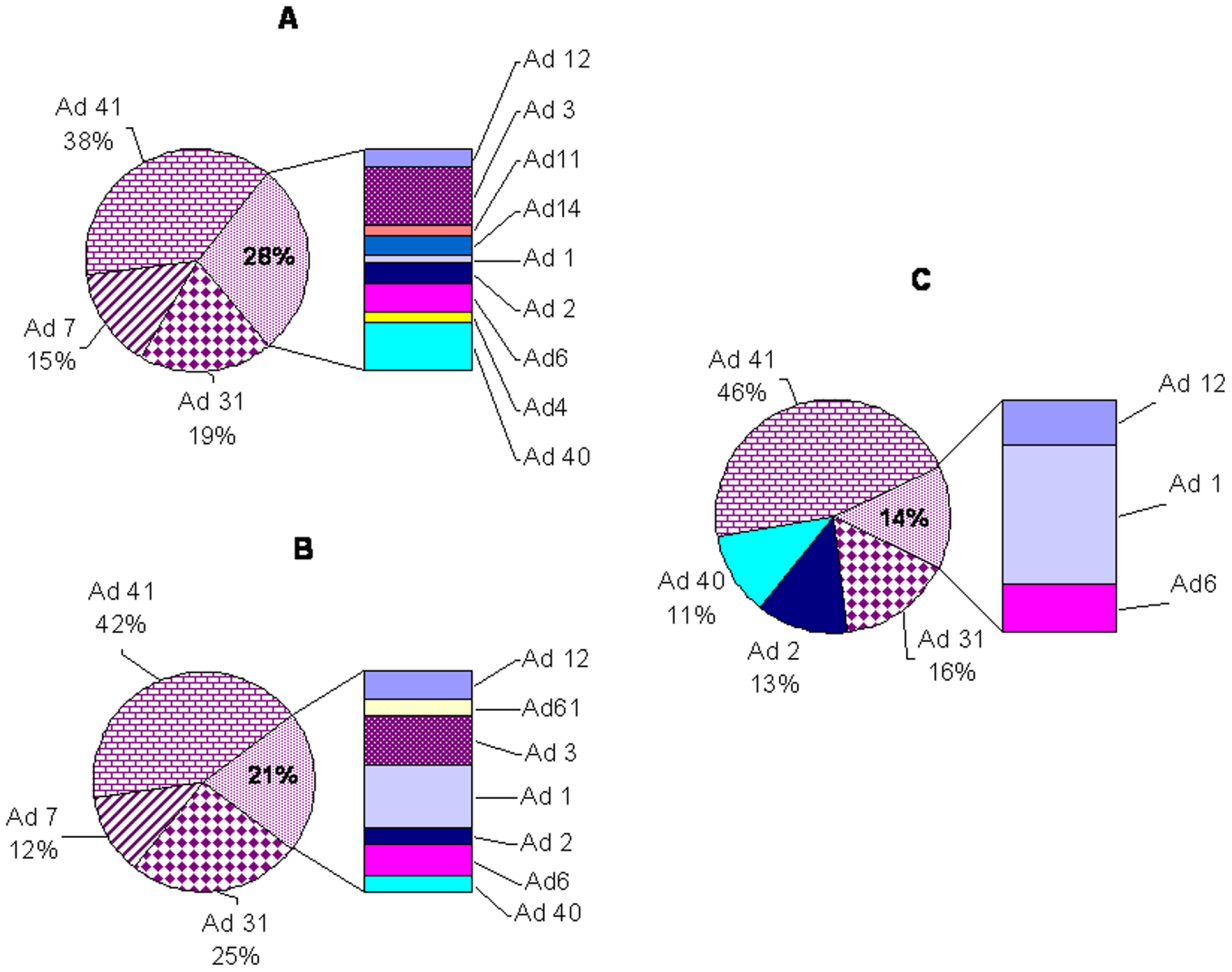
The proportions of adenovirus types among children with acute diarrhea during 2011–2012. (A) The hospitalized children with CAD (IP-CAD). (B) The hospitalized children with HAD (HAD). (C) The pediatric outpatients for acute diarrhea (OP-CAD).

During the two-year study period, Ads, including groups A, B, C, E, and F, were detected in 149 (10.9%) of 1,371 specimens of the hospitalized cases; 82 (9.3%, 82/885) of these were IP-CAD, and 67 (13.8%, 67/486) cases were HAD. Ads that did not include Ad group B were also found in 70 (8.1%) of the 862 outpatient samples. EAd (i.e., Ad40 and Ad41) was the most frequently detected Ad in the IP-CAD (43.9%, 36/82), HAD (43.3%, 29/67) and OP-CAD (57.1%, 40/70) cases, and Ad41 was the dominant member of group F as shown in Figure 1. Non-enteric Ad types were also detected, and the most commonly found of these types were Ad2, Ad7 and Ad31 (Figure 1). Co-infection with rotavirus occurred in 1.1% (10/885), 1.9% (9/486) and 0.6% (5/862) of the cases of IP-CAD, HAD and OP-CAD, respectively, and there was no significant difference between groups (χ^2^ = 4.77, p = 0.092). The rates of detection of Ad among IP-CAD (9.3%) and OP-CAD (8.1%) cases were significantly lower than that among HAD cases (13.8%), and this difference was significant (χ^2^ = 11.76, p = 0.003). Although the difference in the rates of the detection of EAd was not significant among across the IP-CAD (4.1%), HAD (6.0%) and OP-CAD (4.6%) groups (χ^2^ = 2.54, p = 0.281), a significant difference was found in the rates of detection of non-enteric Ad (NEAd) between the IP-CAD (5.2%), HAD (7.8%) and OP-CAD (3.5%) groups (χ^2^ = 12.10, p = 0.002).

### Molecular characterization of Ad 41

**Figure 2:**
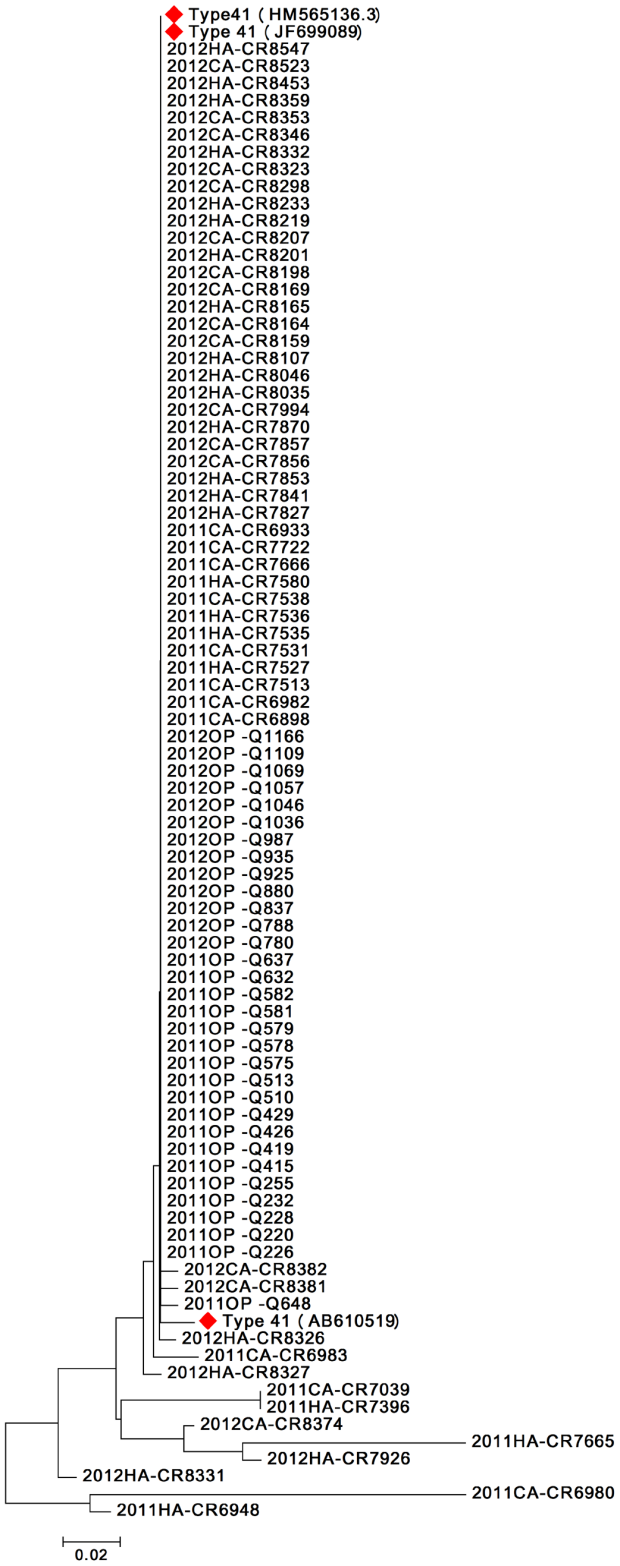
Phylogenetic tree based on partial nucleotide sequences (301 bp) of the hexon gene of Ad 41. 2011 or 2012: the year of detection. CA: community-acquired. HA: hospital-acquired. OP: outpatient. Specimen coding numbers are indicated after the hyphens. Red marks: reference strains, including human Ad 41 hexon gene /Wisconsin isolate HS54/USA/2011 (JF699089), human Ad 41 isolate NIVD103//China/2007(HM565136), and human Ad 41 hexon gene /Tak /Japan /2011 (AB 610519). The hexon nucleotide sequences used in the tree have been deposited under the strain names and accession numbers (in parentheses) as follows: 2011CA-CR6898(JX412892), 2011CA-CR6980(JX412893), 2011CA-CR6982(JX412894), 2011CA-CR6983(JX412895), 2011HA-CR6986(JX412896), 2011HA-CR6988(JX412897), 2011CA-CR6989(JX412898), 2011CA-CR7039(JX412899), 2011CA-CR7145(JX412900), 2011CA-CR7214(JX412901), 2011CA-CR7308(JX412902), 2011HA-CR7396(JX412903), 2011CA-CR7513(JX412904), 2011HA-CR7527(JX412905), 2011CA-CR7531(JX412906), 2011HA-CR7535(JX412907), 2011HA-CR7536(JX412908), 2011CA-CR7538(JX412909), 2011HA-CR7580(JX412910), 2011HA-CR7665(JX412911), 2011CA-CR7666(JX412912), 2011CA-CR7722(JX412913), 2011CA-CR6933(KC953628), 2011HA-CR6948(KC953629), 2012HA-CR7827(KC953630), 2012HA-CR7841(KC953631), 2012HA-CR7853(KC953632), 2012CA-CR7856(KC953633), 2012CA-CR7857(KC953634), 2012HA-CR7870(KC953635), 2012HA-CR7926(KC953636), 2012CA-CR7994(KC953637), 2012HA-CR8035(KC953638), 2012HA-CR8046(KC953639), 2012HA-CR8107(KC953640), 2012CA-CR8159(KC953641), 2012CA-CR8164(KC953642), 2012HA-CR8165(KC953643), 2012CA-CR8169(KC953644), 2012CA-CR8198(KC953645), 2012HA-CR8201(KC953646), 2012CA-CR8207(KC953647), 2012HA-CR8219(KC953648), 2012HA-CR8233(KC953649), 2012CA-CR8298(KC953650), 2012CA-CR8323(KC953651), 2012HA-CR8326(KC953652), 2012HA-CR8327(KC953653), 2012HA-CR8331(KC953654), 2012HA-CR8332(KC953655), 2012CA-CR8346(KC953656), 2012CA-CR8353(KC953657), 2012HA-CR8359(KC953658), 2012CA-CR8374(KC953659), 2012CA-CR8381(KC953660), 2012CA-CR8382(KC953661), 2012HA-CR8453(KC953662), 2012CA-CR8523(KC953663), 2012HA-CR8547(KC953664), 2011OP-Q220(KF669114), 2011OP-Q226(KF669115), 2011OP-Q228(KF669116), 2011OP-Q232(KF669117), 2011OP-Q255(KF669118), 2011OP-Q415(KF669119), 2011OP-Q419(KF669120), 2011OP-Q426(KF669121), 2011OP-Q429(KF669122), 2011OP-Q510(KF669123), 2011OP-Q513(KF669124), 2011OP-Q575(KF669125), 2011OP-Q578(KF669126), 2011OP-Q579(KF669127), 2011OP-Q581(KF669128), 2011OP-Q582(KF669129), 2011OP-Q632(KF669130), 2011OP-Q637(KF669131), 2011OP-Q648(KF669132), 2012OP-Q780(KF669133), 2012OP-Q788(KF669134), 2012OP-Q837(KF669135), 2012OP-Q880(KF669136), 2012OP-Q925(KF669137), 2012OP-Q935(KF669138), 2012OP-Q987(KF669139), 2012OP-Q1036(KF669140), 2012OP-Q1046(KF6691410), 2012OP-Q1057(KF669142), 2012OP-Q1069(KF669143), 2012OP-Q1109(KF669144), 2012OP-Q1166(KF669145)

Of the 219 Ad-positive fecal specimens, 91 were identified as Ad41-positive (41.6%). Phylogenetic analysis revealed that most of Ad41 strains from these specimens were within one genus cluster, which was detected in each of the groups of this study (i.e., IP-CAD, HAD and OP-CAD). These strains shared nearly identical nucleotide sequences with reference strains that have been found in other human populations, and this was particularly true of the Ad41 strains found in the OP-CAD group (Figure 2).

### Gender differences in Ad detection rates

As shown in Table 2, specimens were collected from 1412 boys and 821 girls. The Ad detection rates were 10.4% (147/1412) in boys and 8.8% (72/821) in girls. The rates of Ad detection were similar between genders in each of the groups: IP-CAD (χ^2^ = 0.07,p = 0.785), HAD (χ^2^ = 0.36,p = 0.550), and OP-CAD (χ^2^ = 1.60,p = 0.206). No significant differences in the Ad-positive rates were found between the IP-CAD, HAD and OP-CAD girls (χ^2^ = 4.84, p = 0.089). Interestingly, the rates of Ad detection in the HAD (14.5%) boys was higher than those in the IP-CAD (9.5%) or OP-CAD (9.0%) boys (χ^2^ = 7.30, p = 0.026).

**Table 2:** Gender distributions of Ad positive specimens.

	Boys	Girls	χ2	p
	P (N)*	P (N)		
IP-CAD	51(538)	31(347)	0.07	0.785
HAD	46(318)	21(168)	0.36	0.550
OP-CAD	50(556)	20(306)	1.60	0.206
χ2	***7.30***	4.84		
p	***0.026***	0.089		

*P = Ad-positive cases; N = total cases.

### Ad detection rates in different age groups

**Figure 3:**
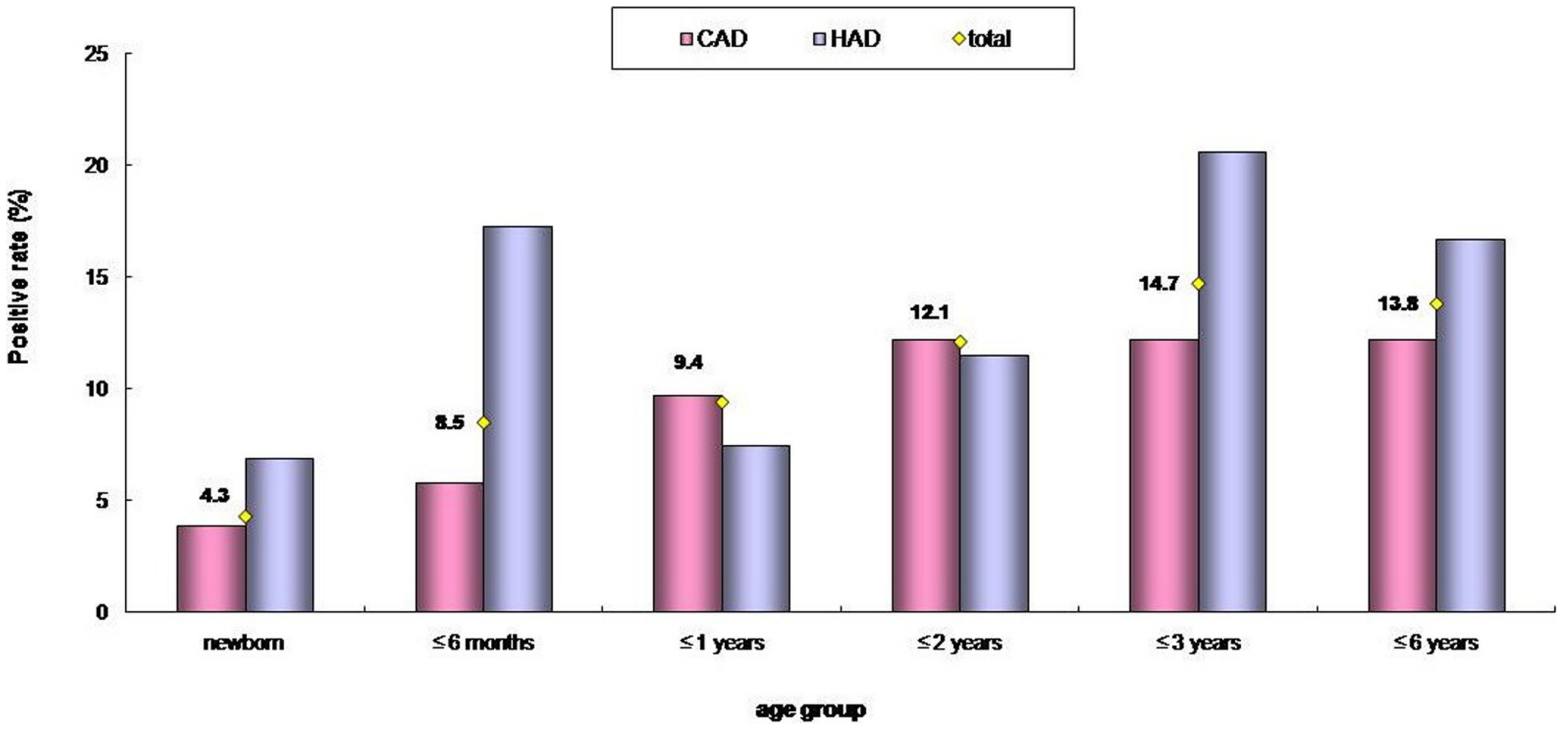
Age distributions of Ad detection rates in children with CAD and HAD. CAD including hospitalized children with CAD (IP-CAD) and outpatient children with CAD (OP-CAD). HAD refer to hospitalized children with HAD (HAD). The range of ages for each age group are indicated in the parentheses as follows: newborn (0–28 days); ≤6 months (28 days–6 months); ≤1 year (6 months–1 year); ≤2 years (1 year–2 years); ≤3years (2 years–3 years); ≤6 years (3 year–6 years).

The numbers of cases in several of the age groups were too low for statistical analyses within the IP-CAD, HAD and OP-CAD groups, but statistical analyses were performed between children with CAD and HAD. As shown in Figure 3, the Ad detection rates in children with CAD were significantly different across six age groups (χ^2^ = 20.39, p = 0.01). Chi-square tests between each pair of age groups revealed that the positive rate of neonates (3.9%) and the ≤6-month-old group (5.8%) were lower than those of any other older age groups, which exhibited detections rates of 9.7%, 12.2%, 12.2% and 12.2%. However, among the children with HAD, no differences in detection rates according to age were found (χ^2^ = 7.86, p = 0.164). Significant differences in the Ad-positive rates between the CAD and HAD children were only found in the ≤6-month-old group (χ^2^ = 23.47,p<0.001) in which Ad-detection rate in infants with HAD (17.3%) was much higher than that in children with CAD (5.8%).

### Monthly distribution

**Figure 4:**
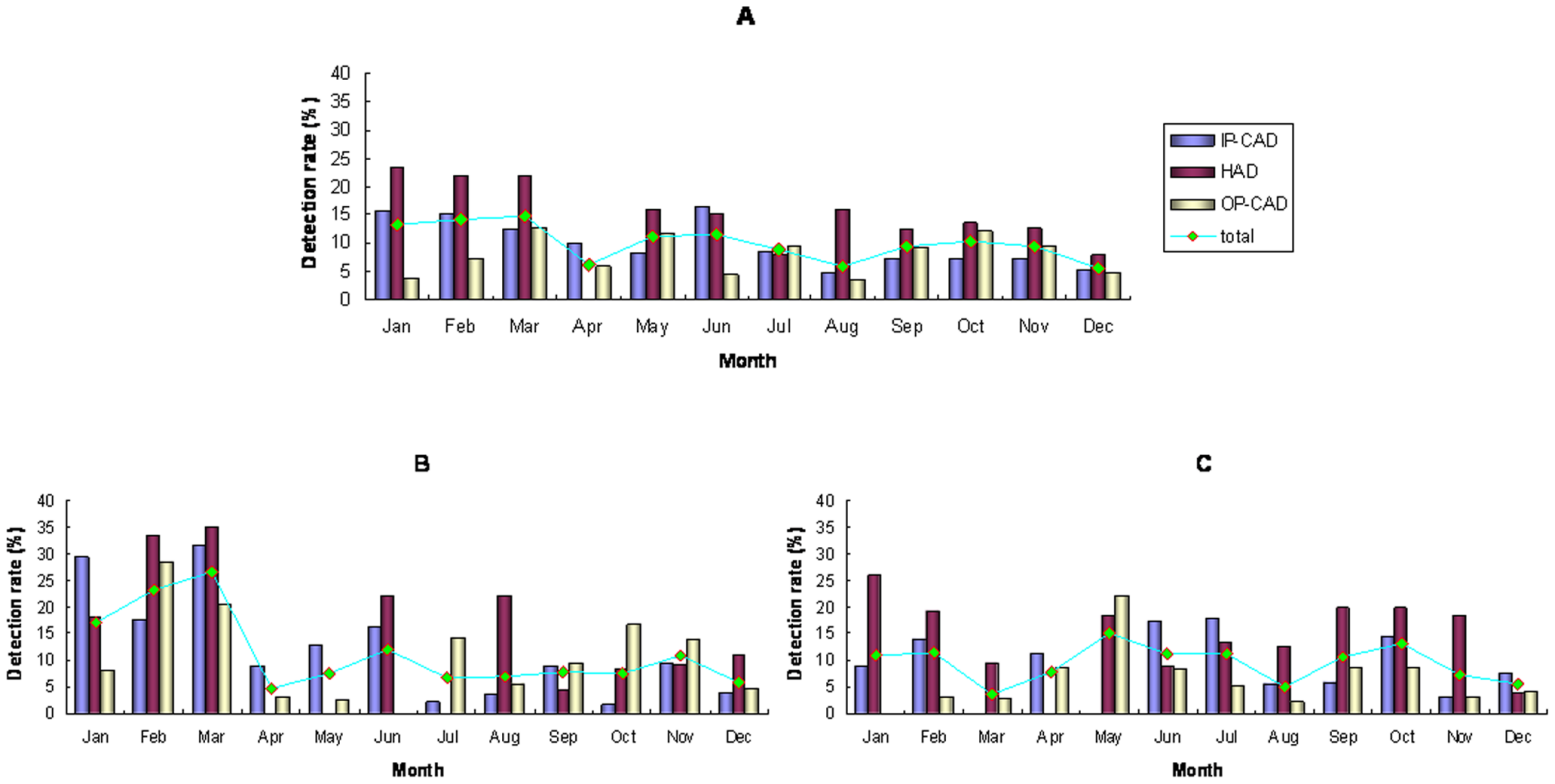
Monthly distribution of Ad detection rates in diarrheal children. (A): Monthly distribution of Ad-positive rates from 2011 to 2012. (B) Monthly distribution of Ad-positive rates in 2011. (C) Monthly distribution of Ad-positive rates in 2012.

In this study, Ads were detected throughout the year, and the monthly distribution of the Ad-positive cases indicated that the highest rates of Ad detection were in January (13.3%, 18/135), February (14.3%, 18/121) and March (14.9%, 25/168). The lowest rates of Ad detection (5.7–6.2%) occurred in April, August and December. However, the seasonal patterns of Ad infection were quite different between the IP-CAD, HAD and OP-CAD children from 2011 to 2012. In August of both years, the detection rats of IP-CAD (3.6% and 5.7%) and OP-CAD (5.7% and 2.2%) were low (Figure 4).

## Discussion

Ads are classified into seven sub-groups and 57 serotypes; one-third of Ads are associated with human diseases [17]. Ads are an important etiological agent of infantile and childhood diarrhea worldwide. In our study, Ads were detected in approximately 10% of the specimens from children with acute diarrhea, and only one-tenth of these specimens also harbored rotavirus. Compared to most previous research, the diarrheal children enrolled in this study were more representative of the population. The hospitalized children were admitted not only for acute diarrhea but also for other reasons such as respiratory diseases, neurological disorders, cardiovascular diseases, renal diseases, etc. These children were divided into CAD and HAD groups according to the time of the onset of diarrhea, which is an internationally accepted definition. The children who visited the outpatient department of our hospital for acute diarrhea were considered to be children with CAD because the diarrhea was their main symptom, but they were not admitted to the hospital. IP-CAD, HAD and OP-CAD groups were created to investigate prevalence characteristics of Ad-positive cases such as detection rates, gender, age, and monthly distributions.

Similar to other reports [7], [18], [19], Ad41 was found to be predominant in this study. No significant differences in EAd-positive rates between the IP-CAD, HAD and OP-CAD groups were found, which is consistent with several other reports [20], [21], [22]. The proportion of NEAd cases has been reported to be 12-43% in several studies [7], [20], [23]; however, we found a NEAd detection rate that exceeded 50% among hospitalized children (IP-CAD and HAD). Ads 3, 7, 31 were found to be the major types in our research, and these groups accounted for 6.0%, 13.4% and 22.2% of all Ad-positive cases, respectively. Approximately 42.9% of the Ad-positive cases were detected as NEAd in the OP-CAD group, and the top three serotypes were Ad1 (8.6%), Ad2 (12.9%), and Ad31 (15.7%). Notably, only Ad 31 was found in both in- and outpatient children, which suggests that Ad31 may be another Ad serotype that leads to diarrhea.

The positive cases of Ad3 and Ad7 were identified in the hospitalized diarrheal children whose admitting diagnoses were primarily respiratory tract infections. Moreover, we found that Ad3 or Ad7 were simultaneously identified in the stool and respiratory specimens of several cases. The serotypes of Ad found in the respiratory and stool specimens of these patients were identical as determined by sequencing the complete fiber genes (data not shown). These data indicated that the difference of Ad detection rate across the IP-CAD, HAD and OP-CAD groups was primarily due to NEAd. The molecular and clinical data mentioned above strongly support the notion that NEAd might be closely related to infantile diarrhea and that its role in the incidence of diarrhea may be more significant in hospitalized children than in outpatient children.

Consist with the findings of other studies [20], [21], [22], [24], [25], our results showed that gender does not play a role in adenovirus infection. Interestingly, analyses of the rates of Ad among hospitalized children with CAD in different ages revealed that the specimens from the children younger than six months of age had a lower Ad-positive rate (5.7%) than did the other age groups. This pattern was not found for rotavirus infection, which occurred indiscriminately across ages in this (data not shown) as has previously been reported [26], [27]. Numerous other studies of Ad-associated diarrhea [10], [20], [28], [29] have only reported the age of highest incidence of Ad infection. Maternal antibodies may play a role in prevention of Ad infections in infants under six months old.

Although we found no evidence for seasonal variation in Ad detection rates in this study, we observed a low prevalence of Ad in August. The rates of EAd in children with CAD were particularly reduced; in 2011 and 2012, these rates were 3.57% and 0 for IP-CAD and 0 and 2.2% for OP-CAD, respectively. Our results, together with reports from Lanzhou, China [20], Brazil [28] and Australia [29], provide strong evidence that, in a variety of populations, adenovirus infection has no consistent seasonal pattern.

The incubation period of Ad is 2 to 14 days, and Ad can be exuviated for months without any symptoms; thus, it is difficult to define Ad-positive cases as nosocomial infections in children with HAD. We only described the characteristics of the Ads that were detected in children with CAD or HAD. To obtain more reliable and meaningful data, a two-year (2011-2012) continuous investigation was performed after a single year (2010) of data had been obtained from hospitalized diarrheal children, and, in the same period, pediatric outpatients with acute diarrhea were enrolled in this study. The subsequent results further confirmed the previous findings [12] by providing more molecular epidemiological evidence and information regarding clinical features than have been provided in the published literature. Therefore, these results should aid our understanding of the role of Ad in hospitalized children with diarrhea, especially those with HAD [10], [11].
